# Transient electromagnetic imaging of saltwater intrusion at the shrinking Dead Sea

**DOI:** 10.1038/s41598-025-15189-0

**Published:** 2025-08-18

**Authors:** Jafar Abu Rajab, Pritam Yogeshwar, Bülent Tezkan, Djamil Al-Halbouni

**Affiliations:** 1 Department of Earth Sciences and Environment, Prince El-Hassan Bin Talal Faculty for Natural Resources and Environment, The Hashemite University, Zarqa, 13133 Jordan; 2 Institute of Geophysics and Meteorology, University of Cologne, Pohligstraße 3, 50969 Cologne, Germany; 3 LIAG Institute for Applied Geophysics, Stilleweg 2, 30655 Hanover, Germany; 4 Institute for Earth System Science and Remote Sensing, University of Leipzig, Talstr. 35, 04103 Leipzig, Germany

**Keywords:** Dead Sea, Transient electromagnetic, Saltwater intrusion, Sinkholes, Ghor Al-Haditha, Jordan, Environmental sciences, Natural hazards, Solid Earth sciences, Geophysics

## Abstract

The Dead Sea (DS) area faces critical environmental challenges, including saltwater intrusion (SWI), widespread sinkhole formation, and topographic changes, largely driven by declining DS water levels. These hazards adversely affect the region’s stability, hydrosystems, and agricultural facilities. In particular, the Ghor Al-Haditha (GAH) region in southern DS has been severely affected by these challenges. This study focuses on imaging saltwater intrusion pathways and their relationship with structural and hydrological features in the GAH region using the transient electromagnetic (TEM) method. A total of 195 TEM soundings of single-turn loop were conducted, spatially covering an area of 4 × 3 km² with a focus along three key stream channel profiles. The data are interpreted using 1D Occam and Marquardt-Levenberg inversion methods. Results are presented as spatial resistivity models at various depths, complemented by interpreted cross-sections for detailed analysis. The derived subsurface resistivity models reveal a saltwater interface with resistivity values less than 1.0 Ωm, detected at 100 m depth and following subsurface stream channels in the area. The main SWI extends 1.75 km inland in the shallow aquifer, most clearly along a well-defined channel in the central part of the study area and serving as a proxy for illustrating the significance of known and hidden hydrogeological pathways in this region, where higher intrusion rates are observed. Additionally, minor anomalies near fault and concealed fault zones may suggest localized upwelling linked to deeper saltwater migration. At the scale of the geophysical survey, the SWI predominantly encompasses the sinkhole belt, while spatially, it appears to be constrained by two bounding stream systems to the north and south. The mid-region resistivity model highlights a stratified subsurface structure comprising freshwater, brackish, and brine zones, emphasizing the model’s value in understanding aquifer vulnerability and guiding water management strategies in the GAH area.

## Introduction

**Fig. 1:**
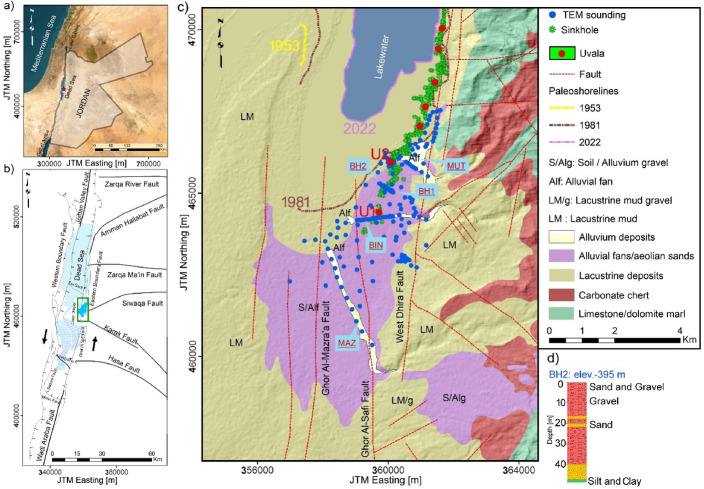
(**a**) Regional map of the Dead Sea; (**b**) Structural features in the Dead Sea Basin (DSB) (modified after^11,12^, Reproduced with permission from the copyright holder. This image is published under a Creative Commons Attribution (CC BY 4.0) license; (**c**) Geological and structural framework of the Ghor Al-Haditha (GAH) study area, modified after Khalil^13^. Key features include the alluvial stream channels: Wadi Mutayl (MUT), Wadi Ibn Hamad (BIN), and Wadi Al-Mazra’a (MAZ). Blue dots indicate the locations of TEM stations, and green dots show the locations of sinkholes. The major sinkhole clusters, uvala 1 (U1) and uvala 2 (U2), are marked by red points. The 1981 and 2022 shorelines are also shown; (**d**) Litho-log of BH2. See borehole area including BH1 and BH2, modified after El-Isa^14^. All maps were generated using ArcGIS Desktop v10.8.2 (Esri, https://desktop.arcgis.com). All spatial data in the maps are projected using the Jordan Transverse Mercator (JTM) coordinate system, which is a national adaptation of the Universal Transverse Mercator (UTM) system. Coordinates are expressed in meters. Conversion between the two grids is straightforward using the right setting and tools.

The Dead Sea (DS), located in the Levant region of the Eastern Mediterranean, is a terminal lake of the Jordan River system in the Dead Sea Basin (DSB) (Fig. 1a). It represents two distinct extreme features, being the largest hypersaline body of water (The water salinity is 340 g/l) and the lowest lake on Earth; as of 2023, its level was approximately 437 m below mean sea level^1^. It is characterized by an arid to semi-arid environment with an average annual precipitation of 70–100 mm^2^.

Two main factors contributed to the significant decline in the DS level (referred to lake water in this research) over several decades: the extensive extraction of minerals from DS water by Jordanian and Israeli industries, and elevated evaporation rates, which were accompanied by the diversion of substantial quantities of freshwater from Lake Galilee and the Jordan River. Originally, the annual water flow in the Jordan River has diminished from 1.5 billion m^3^ in the 1960s to less than 100 million m^3^^3^. Consequently, a drop in the groundwater level is also associated with the decline in the DS level. The lake level has been decreasing at a rate of approximately 0.5 m/year since the 1960s, further changing the hydrological and geological dynamics of the region^4^. As of most recent measurement, the rate of decline in the research region has significantly increased to approximately ~ 1.1 m/year^5^. The significant decrease in the lake’s base level has impacted the geomorphology of the lake’s shoreline region, leading to a significant incision of new water channels^4–6^, as well as the ongoing formation of thousands of sinkholes and landslide triggering^7,8^ (Fig. 1c). In general, the region continues to be vulnerable to active sinkhole hazards, and sinkholes will undoubtedly affect other areas in the future. Several studies have depicted the hazardous effect on the DS coast area infrastructure, i.e., resorts, hotels, and agriculture areas^9,10^. These sinkholes migrate mainly lakeward over time, roughly in line with coastal retreat^5^. Nevertheless, no sustainable engineering solution to this issue is feasible^10^.

The study of saltwater intrusion (SWI) particularly mapping fresh-saline interface (mentioned in this research as saltwater interface) has been widely conducted in coastal regions^15–21^. Though, research on this phenomenon near very saline lakes—particularly in arid and semi-arid environments— remains limited^6,22,23^. Direct methods for determining the saltwater interface include quantitative measurements using groundwater density (based on the Gyben-Herzberg relationship), and chemical analysis such as total dissolved solids (TDS) and groundwater electrical conductivity^6,7,21,24,25^. These methods, while relatively expensive, are spatially limited when the number of monitoring wells is insufficient. However, they ultimately provide valuable insight into the inland extent of SWI; they remain incomplete for detailed studies, particularly in complex environments like DS shorelines. Ghor Al-Haditha (GAH) area to the eastern side of the DS (Fig. 1b), had faced a typical process of SWI, where denser saltwater migrates coastal aquifers in a wedge-shaped pattern, displacing freshwater. This process has been hypothesized to reversed; with the saltwater interface now retreating lakeward^5,26^. This reverse is driven by the continuous decline in the lakewater level, which exposes salt deposits previously submerged in the brine to fresh or brackish water^27^.

From the initial emergence of sinkholes in the early 1980s, the GAH region has undergone numerous geophysical studies^8,14,27–29^. The time domain electromagnetic (TEM) method has demonstrated optimal spatial resolution, making it an efficient tool for mapping subsurface resistivity variations, such as identifying zones of SWI and classifying aquifer salinity^15,20,28,30–33^. Earlier, the TEM method was used for exploring groundwater salinity in the DS area, where aquifer resistivity can be resolved within a range of fractions of Ωm and thereby reducing interpretational model ambiguity^23,27^. In fact, the slower diffusion of electromagnetic fields in highly conductive media, such as saline or brine aquifers, enhances layer resolution, which is crucial for distinguishing conductive groundwater zones. In contrast, rapid field diffusion in less conductive formation limits resolution. Particularly in spatially confined areas like rural settlements in coastal regions, TEM offers a superior depth resolution ratio with small transmitter-receiver layouts, making it more advantageous than most controlled-source electromagnetic (CSEM) and vertical electrical sounding (VES) methods^27,34–36^.

The GAH region has been proposed for consideration as a UNESCO Global Geopark (UGGp) in Jordan, though the designation process is still in its preliminary stages^37,38^. This proposal highlights its distinctive geomorphological and sedimentological features, such as sinkholes, stream channels, wadis, uvala— surface karst expressions formed in areas with soluble salt deposits and lacustrine mudflats (Fig. 1c). This initiative emphasizes the integration of geophysical exploration with sustainable tourism and hazard education to attract visitors, raise awareness of natural risks, and promote environmental education. In this context, TEM method—alongside other geophysical techniques—play a critical role in supporting hazard monitoring and groundwater salinity mapping. There are hundreds of publications in the DS area focusing on different aspects of earth sciences, including geology, geophysics, hydrology, climate, and geomorphology. The early works of authors^6,22,34,39^ provide foundational insights into the localized characteristics of SWI in the DS region.

However, to date, there is no clear understanding or explanation of the spatial SWI pathways in the GAH region or in comparable geological settings in the DS region particularly in shallow clastic aquifers, their inland extent, and their relationship to active sinkhole belt. The current SWI may represent a relict phenomenon, shaped by the historical positions of the DS shoreline. In our study, we address this long-term research gap by connecting the SWI patterns to hydrological pathways and subsurface geological features using TEM method. Accordingly, the main objectives of our study are:

(1) To detect and map the SWI in the GAH region using TEM method, with a particular focus on the main stream channels: the northern Mutayl (MUT), Ibn Hamad (BIN) in the middle, and the southern Al-Mazra’a (MAZ) (Fig. 1c),

(2) To investigate spatially the relationship between SWI pathways and geological structures, including sinkholes, faults, and fluvial channels, (3) To provide details about aquifers’ types and depositional structures at large-scale depression site known as uvala 1 (U1, see location in Fig. 1c).

The paper is organized as follows: the first part presents the geological and hydrological setting. In the second part, we describe the TEM survey design and data processing, including data transformation, error analysis, and data quality. The third part presents and discusses the application of 1D inversion techniques to recover subsurface resistivity variations, resolve potential geological layers, and design interpreted resistivity cross-sections. Finally, we integrate the geophysical results with geological and geomorphological features, focusing on the saltwater interface, intrusion pathways, and their relationships with subsurface structures.

## Geological and hydrogeological setting

The Dead Sea Basin (DSB) formed in the Miocene when the Arabian and African plates diverged due to left-lateral motion along the Dead Sea Transform Fault (DSTF) (Fig. 1b). The left-lateral strike-slip fault system, comprising the Jordan Valley Fault to the north and the Wadi Araba Fault to the south^10,40^. The DSTF has a significant impact on the study area, with several fault zones extending NNW-SSE between the Lisan Peninsula and the Ed Dhira plain; of these faults are the West Dhira, Ghor Al-Safi and Ghor Al-Mazra’a strike-slip fault systems covered by the Lisan Formation and by alluvial fans^9,11^ (Fig. 1c).

In the study area (GAH region) according to Khalil^13^, the stratigraphy of shallow strata can be explained as follows: the Lisan formation deposits (Upper Pleistocene–Recent) (Fig. 1b) highlight a large portion of the study area and consist of poorly-sorted, semi-consolidated to unconsolidated sands and gravels interbedded with minor silts and clays, it also consists of mudflat that describes fine-grained lacustrine sediments (Fig. 1c). These shallow deposits are disrupted up by major semi-dry wadis. Pleistocene gravels from rivers and lakes, along with Holocene and more recent sediments such as alluvium and alluvial fan deposits from several wadis, comprise the top layers of the formation. This top layer contains ~ 11 thousand-year-old salt deposits that were documented at several boreholes in the western side of the DS coastal area^1,28^. It is important to note that the salt diapir, located at a depth of 100 m beneath the surface in the Lisan Peninsula, and the Holocene sedimentary salt layers represent two distinct salt structures with differing characteristics and spatial distributions.

Prior comprehensive borehole lithological and geophysical studies along the western and eastern margins of the DS shoreline^1,4,8,27,41^, inferred geological formations and general subsurface soil sequence compiled in Table 1. Table 1 presents the lithological units derived from boreholes and geoelectrical interpretations. This stratigraphic framework serves as a foundation for the further characterization and interpretation of subsurface models. For example, in Fig. 1d, BH2 shows a sequence of clastic materials characterized by alternating layers of sand and gravel, followed by fine-grained silt and clay.

Furthermore, particularly in coastal clastic aquifers as indicated in our study area, formation resistivity (bulk resistivity) measurements of fully saturated fluids provide valuable insights into subsurface water classification and geological conditions^42^. For example, extremely low resistivities (< 1.0 Ωm) are indicative of very saline aquifers (brine aquifers), such as those associated with the hypersaline DS water^8,27^. Resistivities between 1.0 and 3.0 Ωm typically represent saltwater intrusion in normal coastal region^23^, while slightly higher values (e.g., 3.0–10 Ωm) suggest brackish or diluted fresh water conditions^8^. Fresh water, with minimal salinity, is identified by resistivities exceeding 10 Ωm. Additionally, resistivities in the range of 1.0–2.0 Ωm^41^ can be associated with salt layers, which, in some cases, are encapsulated by overlying and underlying brine-saturated silt and clay units with even lower resistivities (~ 0.3 Ωm), potentially acting as protective barriers that limit vertical fluid exchange and slow salt dissolution (Table 1)^41^.

Although boreholes in our study area (BH1 and BH2) have not yet confirmed the presence of a distinct ‘salt layer’, any reference to our study area in the text may pertain to salt-bearing sediments. However, the presence of a salt layer has been confirmed by boreholes located on the western side of the DS^1,28^.

**Table 1:** Description of the possible geoelectrical layers in GAH study area for shallow aquifer^1,4,8,27,41^.

Layer	Lithology	Resistivity [Ωm]	h [m]	z [m]
1	Gravel/sand	≥ 80	20–30	0
2	Sand/clay	2–30	~ 10	>20
3	Brine silt clay	0.3	1–5	>30
4	Salt layer	1–2	1–5	>30
5	Brine silt clay	0.3	1–5	>30
6	Saturated brine sand/clay	< 0.3	-	>30

Within the limited boreholes in the study area, the historic analysis of the spring and wadi system indicated that groundwater is flowing in a western direction. The discharge of water through these wadis and springs has controlled subsurface aquifer systems. Polom et al.^29^ revealed that the alluvial fan in the area primarily dips northwest (i.e., lake-ward), with seismic reflectors typical of an alluvial fan sequence prograding into a lacustrine environment. The two main shallow aquifer systems encountered in the area are the eastern mountains carbonate aquifer of late Cretaceous to early Tertiary age and the lake Quaternary coastal aquifer or superfacial aquifer^13^ (Fig. 1c).

According to annual observations of Taqieddin et al.^10^, the lake level has been fluctuating since 1861, with the recorded drop reaching 41 m (-437 m) as of 2023^1^. Over this period, the lake level has impacted the most varied and largely clastic aquifer of the study area^23^. The exposed clastic material platform exhibits various hydrological systems and karstic characteristics (i.e., evaporite karst), including new springs, stream channels, uvala, landslides, and sinkholes^4,5,8^. The three principal wadi systems, Wadi Ibn Hamad, Wadi Mutayl, and Wadi Al-Mazra’a, serve as the primary fluvial streams supplying the region with intermittent or flash- flood waters from the eastern terrain (Fig. 1c). These channels deposit consist of semi-consolidated to unconsolidated alluvial fan sediments and terminate at the downstream coastline^8^.

The interaction of fluctuating lake levels with diverse hydrological systems, has created conditions promote for sinkhole formation along the DS coastlines. Key sinkhole formation includes piping mechanism, where groundwater flow through subsurface network of conduits within clastic sediments causes subsurface erosion^10,43^. A combined process of physical and chemical erosion, so called subrosion, affecting the thinly interbedded lacustrine deposits of salt and clay^4,8,29^. Another process involves localized dissolution of subsurface salt edges^28,44^, driven by freshwater direct contact to salt layer surface. Additionally, fault systems may play a significant role by enhancing fluid movement and stress redistribution^45^.

## Methodology

The loop source TEM method is an electromagnetic technique designed to resolve conductive structures with proper lateral and vertical resolution^46^. In TEM measurements a strong direct current passed through an ungrounded transmitter loop. At time = 0, the current is abruptly interrupted, which induces decaying eddy currents in the subsurface. A wire loop receiver records the time-varying secondary magnetic field, allowing resistivity at various depths to be calculated. While TEM effectively detects conductive layers at shallow and deep depths, it has limited resolution for resistive layers. A side-by-side loop survey can enhance lateral resolution, although noise from artificial sources remains an interpretation issue^36^.

### TEM survey design and data acquisition

In total, 195 TEM soundings were measured, covering an area of approximately 12 km^2^. The station distance varies from 50 m along key profiles to several hundred meters on a large scale. A subset of 64 soundings was conducted at a quasi-regular station spacing, particularly along key feature areas named MUT, BIN, and MAZ in Fig. 1c. However, other sites were sparsely located due to accessibility constraints, including sinkholes, farms, mud/salt flats, and urban areas. The area between the 2022 shoreline and the 1981 shoreline (Fig. 1c) is mostly inaccessible due to the instability of the mudflat deposits. However, several survey sites were established in the mudflat area to the south (cf. Fig. 1c). Measurements were carried out using the TEMFAST 48 system with a single loop of 50 × 50 m^2^ and partly 25 × 25 m^2^ configuration. The recording time of 4 ms (4.0 × 10 ^–3^ s) acquired over 40-time gates. To ensure sufficient data quality, acquisition parameters were set to 4 A current and a 50-Hz in-built notch filter.

**Fig. 2:**
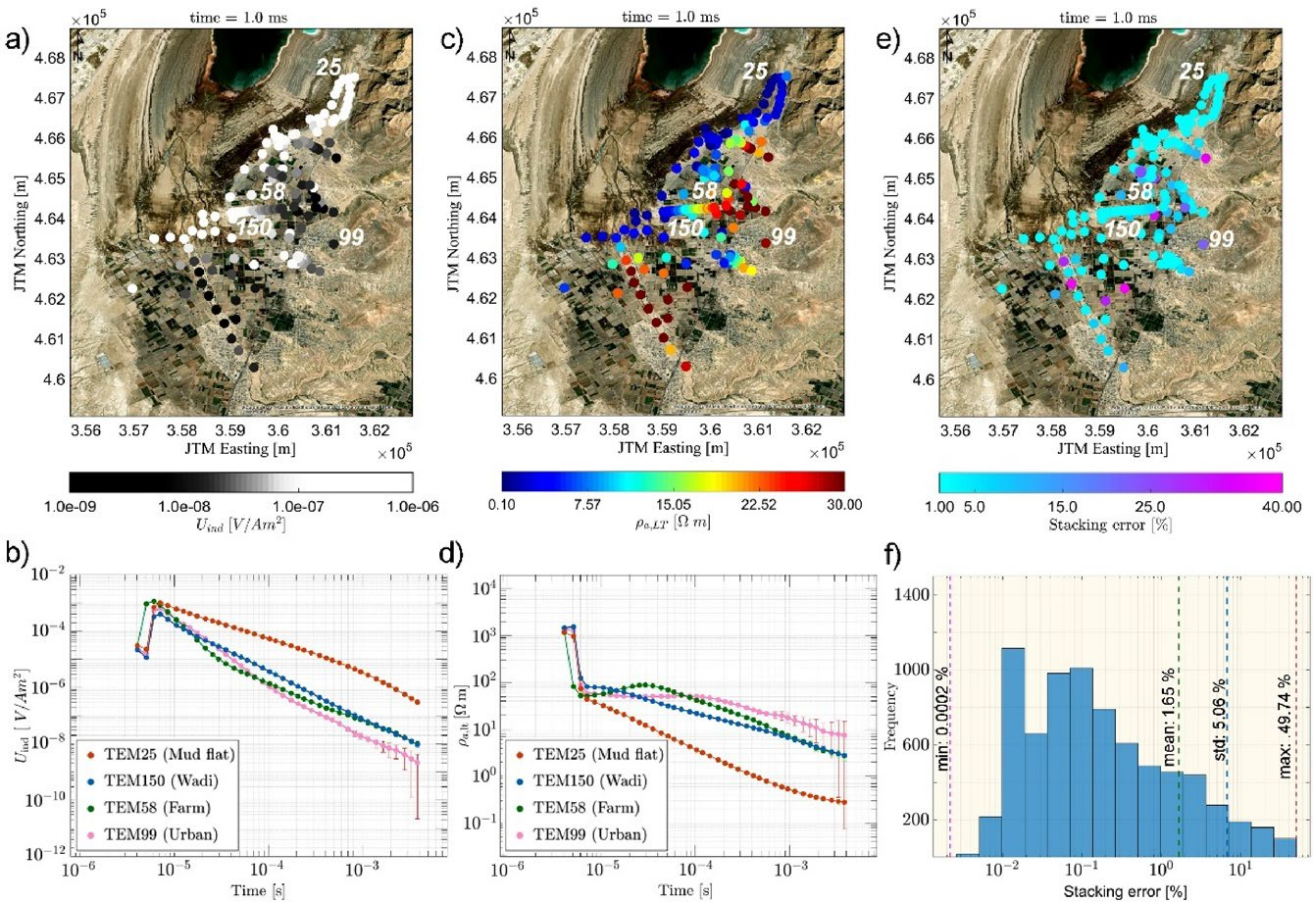
195 TEM sites scatter plot at 1.0 ms as an example (**a**) For induced voltage *U*_ind_; (**b**) The plot of *U*_ind_ vs. time at four geological settings; (**c**) For late-time apparent resistivity $\:{\rho\:}_{L,a}$; (**d**) The plot of $\:{\rho\:}_{L,a}$vs. time at four geological settings. Data points are presented with error bars; (**e**) For stacked error in %; (**f**) Histogram plot of error.

To gain further insights into the data characteristics, we visualize the data spatially for t = 1 ms, which corresponds to intermediate-to-late transient times. Figure 2a and c, & 2e present color-coded maps of the induced voltage *U*_ind_, the late-time apparent resistivity $\:{\rho\:}_{L,a}$, and the TEM data error. We apply the late-time apparent resistivity transformation, according to^47^. Transforming *U*_ind_ to $\:{\rho\:}_{L,a}$reduces the dynamic range and offers an initial impression of the underlying structure^48^. To illustrate variations in decay characteristics, we plot four representative transients corresponding to different subsurface conditions: mudflat (TEM25), wadi (TEM150), farm (TEM58), and urban area (TEM99) (cf. Fig. 1c). The *U*_ind_ map shows fast decays over high-resistive structure, mainly in the eastern parts (e.g., TEM99). Conversely, slow decay dominates the mudflat area (TEM25) (Fig. 2a and b). Between these locations, a notable variation in $\:{\rho\:}_{L,a}$is evident in the sounding curves (cf. Fig. 2c and d), ranging from tens of Ωm in gravel and sand deposits, to less than 1.0 Ωm in the region of the conductive mudflat. Taking a closer look, it appears that the overall patterns of $\:{\rho\:}_{L,a}$ & *U*_ind_ do not follow a clear orientation—i.e., partly the pattern is conductive along the 1981 shoreline, partly it seems coupled to stream channel occurrence, and partly it extends laterally eastwards (Fig. 2a and c).

In general, the error distribution depicted in Fig. 2e presents spatially uncorrelated stacking errors, only few locations indicate noisy data with larger errors at late times. At early times, the first three data points of all transients are distorted due to oscillating current and are therefore excluded (Fig. 2d). Figure 2f presents a histogram of the stacking error for all sounding data. Most stacking errors are below the average value of 1.66%. Errors below 1.0% are not meaningful given the large experimental setup for TEM soundings^36,46,49^. To avoid over-interpretation, a minimum error of 2.0% is chosen for all data, which is in the range of the mean observed stacking error. For example, measurements from the urban site TEM99 show increased noise for observed times greater than 1.0 ms, likely due to electromagnetic coupling with nearby infrastructure (Fig. 2d). It should be noted that the survey was optimized for minimal interference with manmade structures, although some interference remained unavoidable.

Cross-referencing the generated maps with the reference locations (labeled soundings in Fig. 2) reveals that areas along the mudflat deposits, including the area located between BIN and MAZ channels (cf. Fig. 1) are characterizing by high *U*_ind_ (1.0 × 10^− 6^ V∕Am^2^), low $\:{\rho\:}_{a,L}$(< 1.0 Ωm), and low error (< 3.0%), see Fig. 2a and c, & 2d. The farthest eastern parts of MUT, BIN, and MAZ areas have comparatively resistive structure (e.g. 30 Ωm) coincided with low *U*_ind_ (e.g., 1 × 10^− 9^ V∕Am^2^) (cf. Fig. 2b, at t = 1 ms).

### Inversion and model resolution

Conventional 1D inversion techniques were used to interpret the data^32,50^. We apply Occam’s technique using a first-order (R1) and a second-order (R2) smoothness constraint^51^. The Occam’s inversion results are reasonable if no sharp resistivity contrasts are expected. However, modeling a thin layer is difficult without decoupling the smoothness constraint. Subsequently, a damped Marquardt-Levenberg (ML) technique^52^ is used to produce a minimum layered model. The initial model is obtained from prior Occam results. A hybrid approach utilizing ML and Monte-Carlo inversion techniques^50^ is used to derive equivalence models that explain the data equally well within the errors. For each inversion scheme, the error-weighted data misfit $\:\chi\:$ is calculated.

1 $$\:\chi\:=\:\sqrt{\frac{1}{N}\sum\:_{i=1}^{N}{\left(\frac{{d}_{i,obs}-\:{d}_{i,calc}}{\varDelta\:{d}_{i,obs}}\right)}^{2}}$$

where $\:{d}_{i,obs}$ and $\:{d}_{i,calc}$ are the observed and the calculated data, respectively. The data type difference is normalized to measurements errors $\:\varDelta\:{d}_{i,obs}$ of N data point. When the data is optimally fitted and the error estimates are appropriate, $\:\chi\:\approx\:$ 1.0 is expected.

The depth of investigation (DOI) is a key parameter for determining the depth at which reliable sounding interpretation can be achieved. Approaches such as those proposed by Meju^53^ and Spies^54^ are well-established in the literature. Nevertheless, for this study, we adopt a case-sensitive approach, defining the DOI as the depth at which the Occam R1/R2 models diverge, i.e. where the inversion is driven by regularization rather than by the observed data^55,56^.

In terms of the derived model’s parameter resolution, importance values are computed from the singular value decomposition of the Marquardt Jacobian matrix. Importance values indicate parameter resolution: 0.75–1.0 for well-resolved, 0.5–0.75 for moderately resolved, and below 0.5 for poorly resolved parameters^56^.

## Results and discussion

We calibrated TEM models using borehole lithological data, which helped elucidate and validate the interpretation of one-dimensional (1D) subsurface resistivity. Furthermore, we outline the core principles and foundational criteria for detecting and characterizing SWI in the study area, drawing on key insights and findings from the primary literature. In support of this interpretation, we also derived and mapped the DOI to assess the sensitivity and effective penetration depth of the TEM data across the region. Subsequently, the SWI is delineated throughout the GAH region, and also emphasizing principal stream channels. The investigation evaluates the correlation between SWI paths and geological structures, such as sinkholes, faults, and stream channels. Finally, a detailed, small-scale study is conducted on the Ibn Hamad stream channel (BIN, Fig. 1c), where various geological and geomorphological structures are examined in the region of uvala1 (U1).

### Comparison of borehole lithology and TEM models

**Fig. 3:**
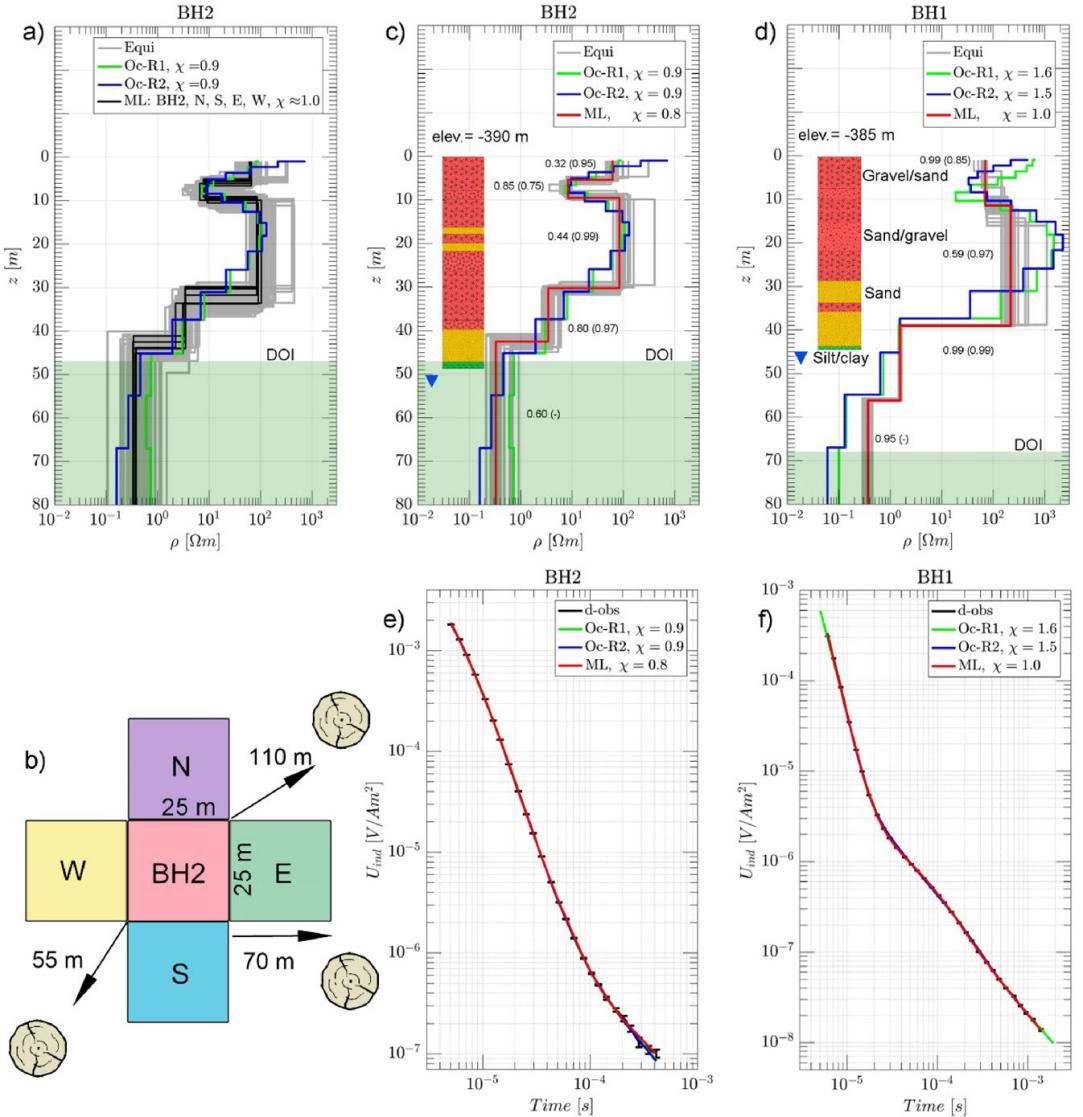
Geoelectrical models of boreholes area (**a**) Five ML models recovered at BH2; (**b**) The survey design consists of five loops centered around the borehole location, with letters indicating geographic directions based on the borehole at the center. The circular symbol represents the orientation and location of the refilled sinkholes, whose sizes are no longer visible in the field but are inferred from historical satellite imagery; (**c**) and (**d**) Present 1D resistivity inversion for Marquardt (ML), Occam R1/R2, and Monte Carlo equivalent results obtained for boreholes BH1 and BH2. Inversion importance values for layer resistivity (in brackets), followed by layer thickness importance. The DS saltwater level at the time of measurement is -437 m and marked by blue triangle; (**e**) and (**f**) Present fitting of observed and calculated data using different inversion approaches.

There are only two boreholes (BH1 and BH2) in the study area, located 340 m apart (cf. location in Fig. 1c). Figure 3 compares the lithological data and 1D models. BH2 is located in the area of a refilled sinkhole (Fig. 3b), raising questions about the validity of the 1D inversion and subsequent interpretation. To assess data consistency and identify potential 3D effects, five soundings with 25 m loop sides were recorded around BH2 (Fig. 3b).

The analysis of the five ML models around BH2, presented in Fig. 3a, reveals consistent resistivity and thickness, suggesting that a 1D layered-earth structure adequately describes the data within the typical induction volume of the TEM sounding. The presence of a refilled cavities near BH2, associated with sinkholes (Fig. 3b and e), do not produce any significant data distortions. While minor lateral variations may exist, the induced currents predominantly flow horizontally, indicating negligible 2D/3D effects in the data^55^. A similar resistivity structure is observed at the BH1 sounding location. Notably, the area near BH1 shows no evidence of sinkhole development.

This observation is consistent with the broader pattern across the study area. The vast majority of the TEM soundings are located in regions with no visible hollow structures; most sinkholes have been filled or naturally refilled over time and no longer present substantial topographic or volumetric contrasts in terms of TEM loop size. Based on our interpretation, supported by simplified forward modeling, these sinkholes fall within the shallow eddy current penetration zone and primarily behave as minor topographic irregularities rather than true 3D resistive bodies capable of distorting late-time TEM responses. Furthermore, the only area with clearly visible and active (hollow) sinkholes (uvala 2 (U2), see Fig. 1c) is situated well beyond the inland extent of the SWI and was modeled using a 1D approach, which aligns with the study’s primary objectives. The overall size, depth, diameter, and morphological relationships of sinkholes in the GAH region are comprehensively described in references^4,5^.

Based on the preceding analyses, the inversion models for BH1 and BH2 delineating four to five layers under the assumption of uniform isotropic resistivity with optimal data fit of χ ≈ 1.0 (Fig. 3e and f). Additionally, the lithology appears to be quite uniform. The model parameters are well resolved with mostly tight equivalent models and laterally consistent model parameters (cf. Fig. 3c and d). The shallow layers of gravel and sand-gravel layers have rather wide resistivity variation (10–200 Ωm) with well-resolved layer thicknesses (imp ≈ 0.95). Within this variability, a distinct conductive layer becomes evident. Owing to their proximity to DS, BH1 and BH2 Occam models exhibit a shallow conductive layer (ρ ≈ 10 Ωm, z < 10 m) within the gravel–sand unit, attributed to the infiltration of shallow, diluted saltwater (Fig. 3c and d). Similar TEM models between boreholes confirm this conductive layer (cf. Fig. 1c); however, these TEM models are not presented in this study. The underlying sand layer (2.0–3.0 Ωm) show a well resolved resistivity and thickness (imp ≈ 0.97). The bottom silt-clay layer is conductive, with a resistivity of 0.3 Ωm, and is moderately to well-resolved, reflecting saturated DS brines at the saltwater level of -437 m, which is marked by a blue triangle in Fig. 3c and d.

DOI estimates obtained using the R1/R2 divergence criterion are presented in Fig. 3c and d. For BH1 and BH2, the DOI values range from 47 m to 67 m, aligning well with Meju’s estimates of 55 m and 63 m^53^, respectively. Therefore, the TEM models effectively detect DS brine-bearing sediments composed of silt and clay (0.3 Ωm) indicative of saturated DS brine and the direct impact of SWI. Notably, the SWI level—indicated by the blue triangle in Fig. 3c and d—coincides with the base of the silt-clay layer, located at 48 m for BH1 and 53 m for BH2 (both at the 2022 DS level of − 437 m) (see Baer et al.^1^. While the overall lithological sequence is consistent across both boreholes, direct correlation with resistivity layers is not always straightforward. Sharp gravel-to-sand transitions, mixed sand–gravel units, and thin interbeds contribute to textural complexity. Combined with salinity variations and partial saturation, these factors significantly affect bulk resistivity. For example, saline-saturated sandy layers may show low resistivity similar to finer silty clay sediments, complicating lithology–resistivity correlation.

### Depth of investigation (DOI) of TEM data

**Fig. 4:**
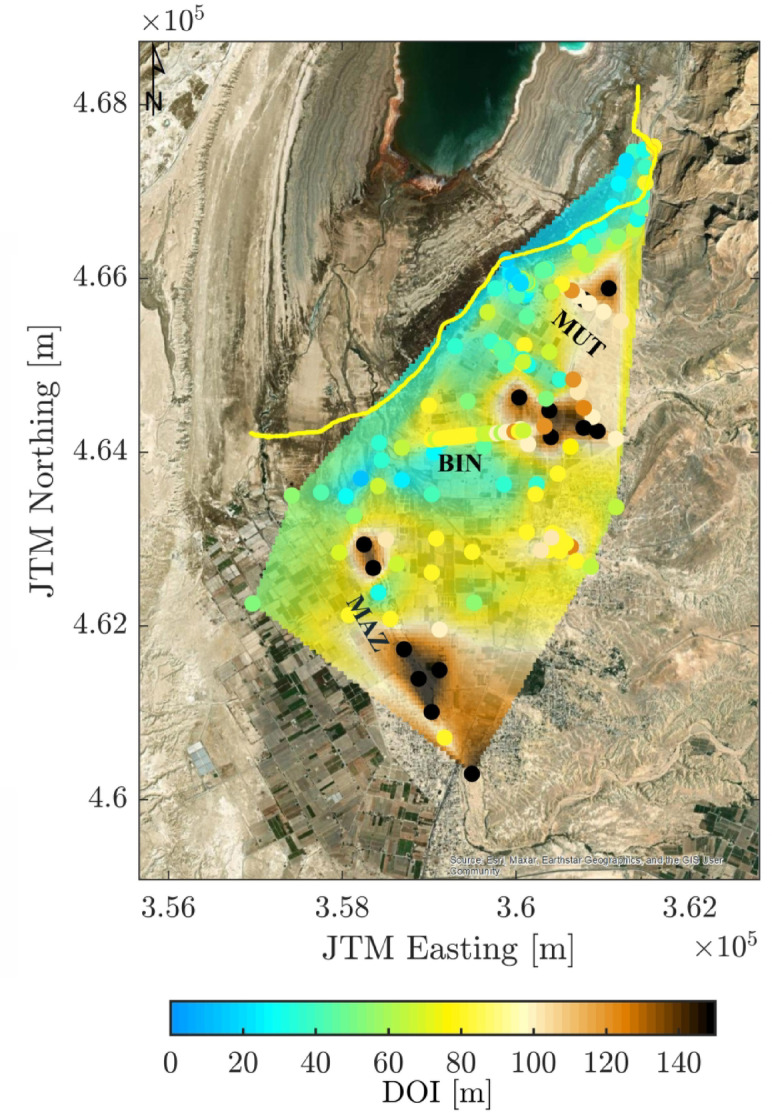
DOI map for 195 TEM sites across the GAH area. The map includes the MUT, BIN, and MAZ stream areas, along with the 1981 DS shoreline (yellow line) for spatial reference.

The DOI values derived from the TEM dataset using the R1/R2 divergence criterion^55,56^ were interpolated over a regular grid using the natural neighbor interpolation method to produce a continuous and spatially coherent map of DOI coverage (Fig. 4). This approach allows for visualization of the varying sensitivity across the study area, reflecting the influence of subsurface electrical properties and terrain conditions.

DOI values are presented at 20 m intervals, illustrating significant spatial variations across the region. Shallower DOI values (< 20 m) are concentrated along the conductive north western margin near the 1981 DS shoreline, where brine-saturated sediments limit the penetration of eddy currents. In contrast, deeper DOI values (up to 150 m) are observed further inland, particularly toward the northeast (BIN and MUT) and southeast in areas associated with the MAZ stream, where coarser and more resistive alluvial deposits are predominant (Fig. 1c).These variations are consistent with the expected effects of SWI and support the subsequent interpretation of subsurface conditions derived from the resistivity models.

### Saltwater intrusion interface: a large-scale analysis

The following analyses bring together depth-based mapping, key resistivity profiles, and boundary detection to examine the saltwater intrusion interface across the GAH region. They reveal how SWI advances through different geological settings, shaped by varying materials and structural conditions.

#### Spatial SWI at several depths

The primary key problem is to effectively detect and spatially characterize SWI in the GAH region, since SWI is present in a complex and heterogeneous environment shaped by materials such as salt, clay, sand, silt, and gravel. Two main criteria are defined.

First, the basic assumption is that bulk resistivity values serve as a reliable indicator of SWI. Bulk resistivity values ranging from 0.55 to 1.0 Ωm specifically and distinctly define the presence of DS brine within the pores of clastic sediments^27^ (cf. Fig. 3c and d, which illustrate the DS level and the resistivity of silt-clay sediments bearing brine, showing values below 1.0 Ωm). Earlier studies have highlighted the utility of bulk resistivity values in delineating zones of varying salinity. Yechieli^6^ used the Ghyben-Herzberg relation to demonstrate that resistivity values near 1.0 Ωm correspond to the saltwater interface. Furthermore, Yechieli et al.^23^ investigated the salinity patterns within aquifers and sub-aquifers of the DS region, identifying three distinct zones: bulk resistivity values below 1.0 Ωm (diluted brines), 1.0–<5.0 Ωm (transition zones), and ≥ 5.0 Ωm (freshwater/brackish water). Similar patterns were also observed by Al-Halbouni et al.^8^.

**Fig. 5:**
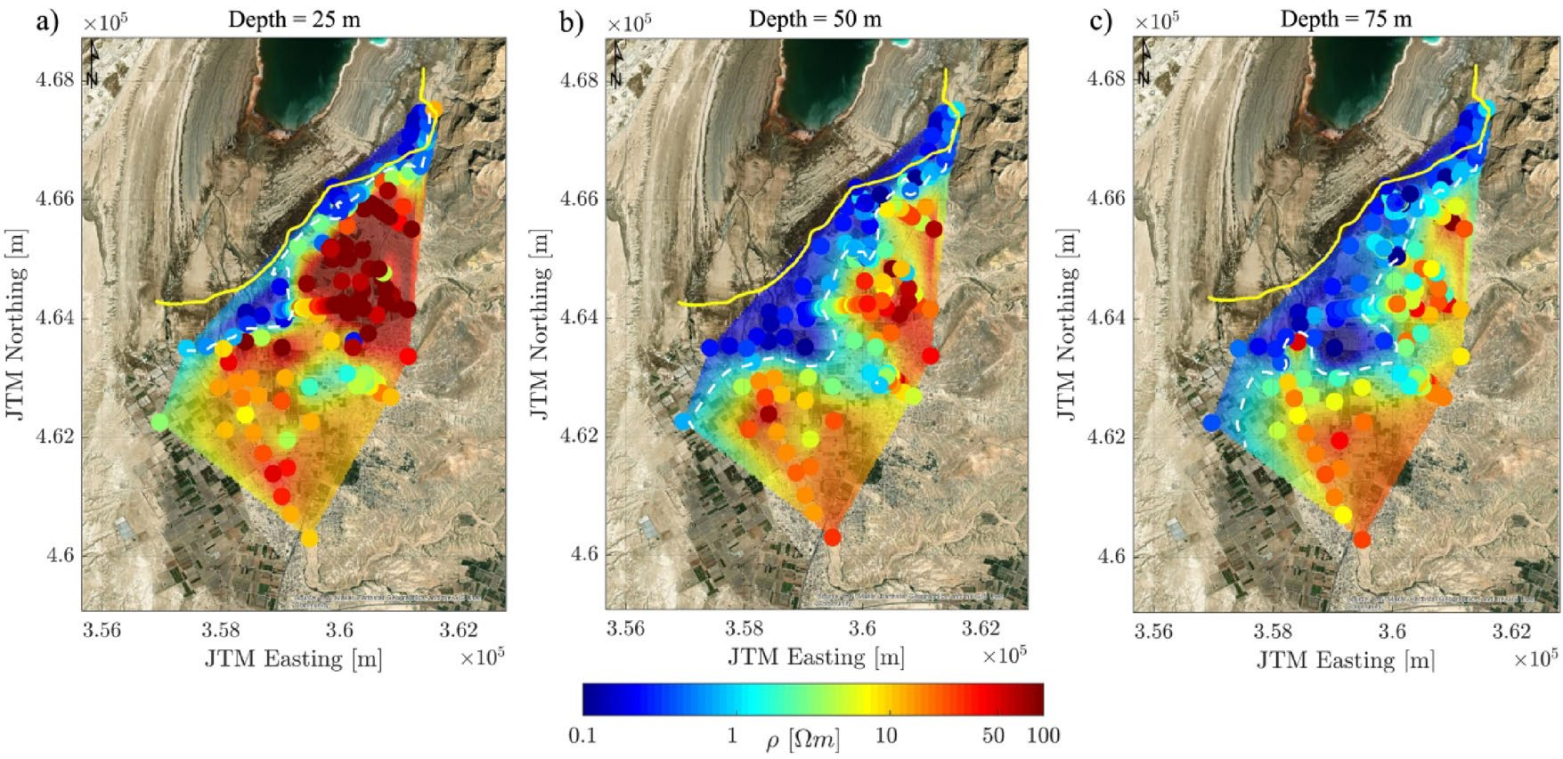
Interpolated maps of Occam R1 resistivity results with scatter plot of data at selected depths: (**a**) z = 25 m; (**b**) z = 50 m; (**c**) z = 75 m. The yellow line indicates the 1981 shoreline, and the dashed white lines quantitatively delineate the SWI boundaries at a resistivity of 1.0 Ωm.

Second, for the purpose of observing such ground vertical and lateral variation and comparison among different regions, we used the reference areas, or benchmark, located in the mudflat area saturated with brine water, with bulk resistivity < 0.3 Ωm according to Yechieli et al.^23^ and Ezersky & Frumkin^27^, i.e., adjacent to the 1981 shoreline (see Fig. 1c and the yellow line presented in Fig. 5). Hence, the 1981 shoreline is considered a key marker for potentially trapped brine water^23^, after which sinkhole development became prominent. This area has since transitioned into a region of mudflat or lacustrine salt flat, extending to the north. Consequently, Fig. 5 illustrates the subsurface resistivity distribution derived from Occam R1 inversion models at depths of 25 m, 50 m, and 75 m, and data were interpolated using the natural neighbor interpolation method. Key observations include:

- Bulk resistivity values below 1.0 Ωm predominantly characterize zones closer to the 1981 shoreline.
- A general decrease in resistivity values is observed with increasing distance from the 1981 shoreline; note that the DOI near the 1981 shoreline is less than 75 m for several soundings.
- Differential and heterogeneous SWI spatial patterns are evident, particularly in the central region around BIN, where the impact of SWI is locally pronounced, contrasting with the relatively uniform spatial patterns observed in the northern and southern regions. This disparity highlights the localized variation in saltwater migration (see DOI in Fig. 4).

#### Analysis of key profiles

**Fig. 6:**
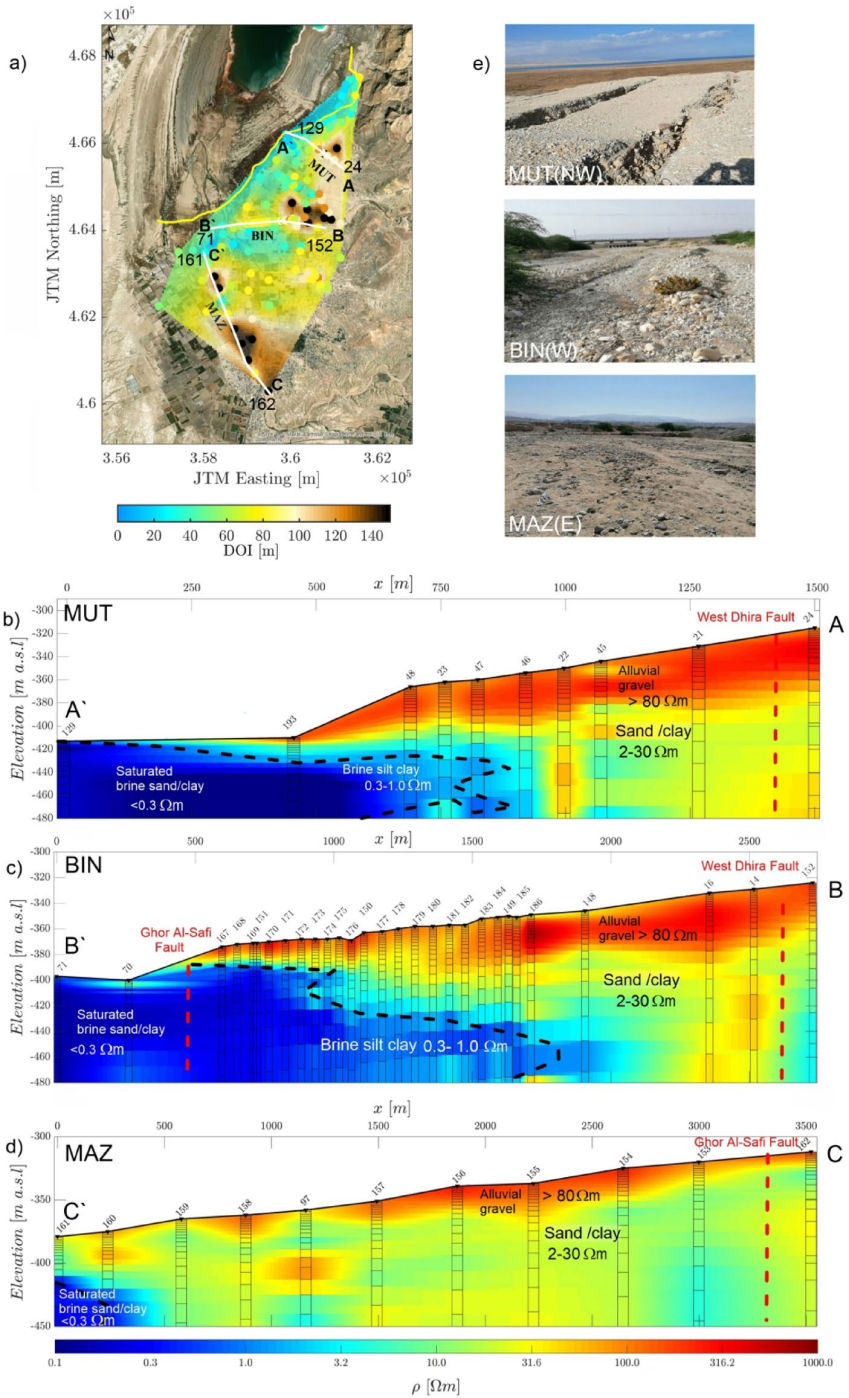
Occam R1 resistivity section of the interpreted saltwater interface along the main key areas (**a**) Profile orientation is presented in the DOI map; (**b**) The 1D resistivity section for Wadi Mutayl (MUT); (**c**) The 1D resistivity section for Wadi Ibn Hamad (BIN); (**d**) The 1D resistivity section for Wadi Al-Mazra’a (MAZ). The dashed black line, indicating a value of less than 1.0 Ωm, tentatively represents the saltwater interface. The global misfit χ is ~ 1.0 for all resistivity sections; (**e**) Field photos representing the main surveyed areas.

To elucidate the heterogeneity in SWI, resistivity cross-sections were constructed using Occam inversion models for three key areas—MUT, BIN, and MAZ (Fig. 1c)—as shown in Fig. 6a. A series of TEM soundings were integrated to generate a quasi-two- dimensional (2D) resistivity model. We applied scattered data interpolation using Delaunay triangulation to improve visualization of continuous subsurface images, highlighting lateral resistivity variations while preserving vertical resolution and inversion integrity without imposing artificial lateral smoothing. These models reference the SWI approximately against the 1981 shoreline to the west, identified at resistivity values below 1.0 Ωm and delineated by a black dashed line embedded in all resistivity models (Fig. 6b–d). We observed that the resistivity structure across all sections consistently transitions through three main layers, numbered from top to bottom: (1) highly resistive uppermost layers of gravel and sand (> 80 Ωm), (2) more conductive layers of sand, silt, and clay (2.0–30 Ωm), and (3) saltwater-saturated deposits near the western margin of the study area (< 0.3 Ωm). These layer numbers are used throughout the text for clarity. Refer to Table 1; Fig. 3 for the resistivity–lithology relationships.

Across all three stream channels, the saltwater interface is generally encountered within the upper 100 m and becomes more pronounced in regions where the ground surface lies lower than − 437 m elevation, which corresponds to the DS level at the time of measurement. This corresponds to zones of unsaturated brine with resistivity values of 0.3–1.0 Ωm that extend farther eastward. Wedge-like interface patterns within the alluvial stream deposits are another common feature, indicating the intrusion’s bending point and extent. Notably, the DOI distribution along the MUT, BIN, and MAZ stream zones (Fig. 6a) supports the interpretation of this interface; in most cases, the delineated SWI boundary lies above the DOI depth, affirming the ability of the inversion models to resolve this transition and lending credibility to the estimated inland extent of the intrusion.

*MUT stream channel*: The resistivity section for MUT shows the SWI confined to a horizontal distance of approximately 0.8 km. This profile is the closest to the present-day shoreline. The structure reveals significant heterogeneity in the uppermost layers due to the influence of a landslide and associated alluvial fans (Fig. 6b and e). These features result in surface cracks and an anomalous structure extending into deeper layers particularly around sounding 45. The West Dhira Fault has a certain influence on shaping this section (cf. Fig. 1c), with its subtle impact observed around soundings 24 and 21, as indicated by the dashed red line. In addition, the deeper resistive structure observed at sounding 22 contrasts with the conductive zone identified around sounding 45 (~ 3.0 Ωm), which may indicate saltwater upwelling along a potential concealed fault zone. This upwelling likely transports saline water from depth toward the surface, contributing to the formation of a vertically extensive conductive zone that may also influence surface features such as cracking^6,8^.

*BIN stream channel*: In the BIN resistivity model, the SWI extends significantly, reaching 1.75 km, making it the most extensive among the three channels. Wedge-like interface pattern is prominent, indicating the eastern boundary of the intrusion (Fig. 6c). Fault influence is evident in this area, where both the West Dhira Fault (between soundings 14 and 152) and the Ghor Al-Safi Fault (near sounding 167) has different imprints on the resistivity structure, as shown in Fig. 6b. According to Yechieli^6^ and Al-Halbouni et al.^8^, the carbonate aquifers on both sides of the Dead Sea—extending eastward in Jordan and westward in Israel—have been affected by faulting, primarily through several normal fault systems. These faults facilitate groundwater upwelling along conduit planes, leading to partial recharge of the overlying alluvial aquifers. Although this recharge remains limited, it may explain the observed low-resistivity anomaly east of the West Dhira Fault, as presented in the BIN resistivity model (see the conductive anomaly of ~ 3.0 Ωm around sounding 152 in Fig. 6c, similar to what has been seen in the MUT model)—possibly supporting the presence of such an upwelling process.

*MAZ stream channel*: The MAZ resistivity model indicates the most restricted extent of SWI, confined to a horizontal distance of 0.25 km. However, a wedge-like intrusion interface is observed in the saturated brine between soundings 160 and 161, though less prominently than in the BIN and MUT models (Fig. 6d). The low resistivity value of ~ 3.0 Ωm at sounding 153 might indicate brackish water, supporting the idea that deeper, fault-controlled aquifers may be involved in the upwelling. Although the Ghor Al-Safi Fault is a strike-slip fault, it may still facilitate vertical fluid movement. This structural influence is consistent with the conceptual model of Yechieli^6^.

The overall structure reflects a homogenous upper layer compared to MUT, with fewer disruptions, for example, near noisy sounding 97 (cf. Fig. 2e), where an anomalous resistive body appears isolated and lacks correlation with the surrounding soundings, possibly due to localized lithological variations or data noise.

#### Depth to the saltwater interface

**Fig. 7:**
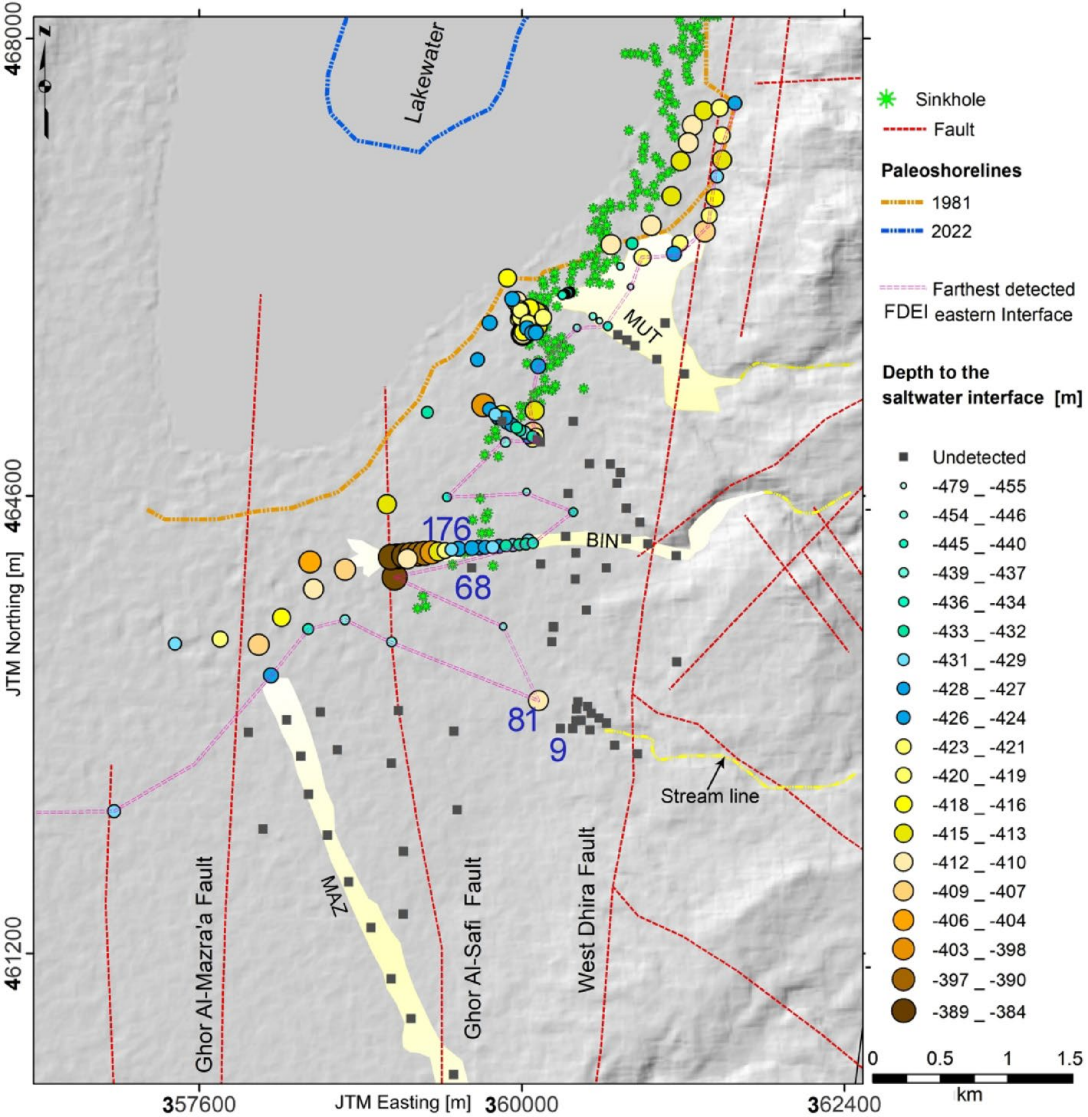
A spatial map illustrates the depth to the saltwater interface, which is embedded within the terrain and the main geological structures. The interface line connects layers of resistivity less than 1.0 Ωm representing DS saline water-bearing sediments (FDEI). Shaded relief topography of the study area obtained from 30 m-resolution SRTM USGS EROS archive data.

To delineate the spatial distribution of the latest saltwater interface in the study area, we identified the depth to the conductive boundary where resistivity values drop below 1.0 Ωm. This depth is assumed to correspond to slightly saturated, brine-bearing sediments, as suggested by Ezersky & Frumkin^27^. Among the 195 TEM soundings, 61 did not detect the saltwater interface (see black squares in Fig. 7), primarily in the eastern and southeastern portions of the study area. The absence of interface in these soundings, including those closely spaced, suggests that the interface is genuinely absent in these areas. This interpretation is supported by the lack of lateral continuity in the intrusion, rather than being attributed to limitations in DOI (see Fig. 4). To delineate the possible current extent of the saltwater interface, we defined a line connecting the farthest detected eastern interface (FDEI) at various depths (pink line in Fig. 7). To delineate the FDEI, we adopted two main criteria:

1. Only soundings with sufficient DOI to resolve the expected interface depth were considered
2. Sites were selected based on the presence of a low-resistivity zone (< 1.0 Ωm) and acceptable misfit ($\:\chi\:$) values across corresponding Occam R1/R2 and ML models, with equivalent models used as constraints. Hence, inversion quality has high importance values for the low resistivity zones.

The FDEI analysis revealed three intrusion patterns. First, the SWI exhibits two bulging trends: (a) one aligned with the BIN structural system, and (b) another occurs along a probable concealed channel located south of and adjacent to the BIN system; see the yellow stream line indicating the concealed channel in Fig. 7. Second, the FDEI encompasses the sinkhole belt across most of the surveyed sites. Third, no significant association between the FDEI and major strike-slip fault systems, such as the Ghor Al-Mazra’a and Ghor Al-Safi faults is observed.

**Fig. 8:**
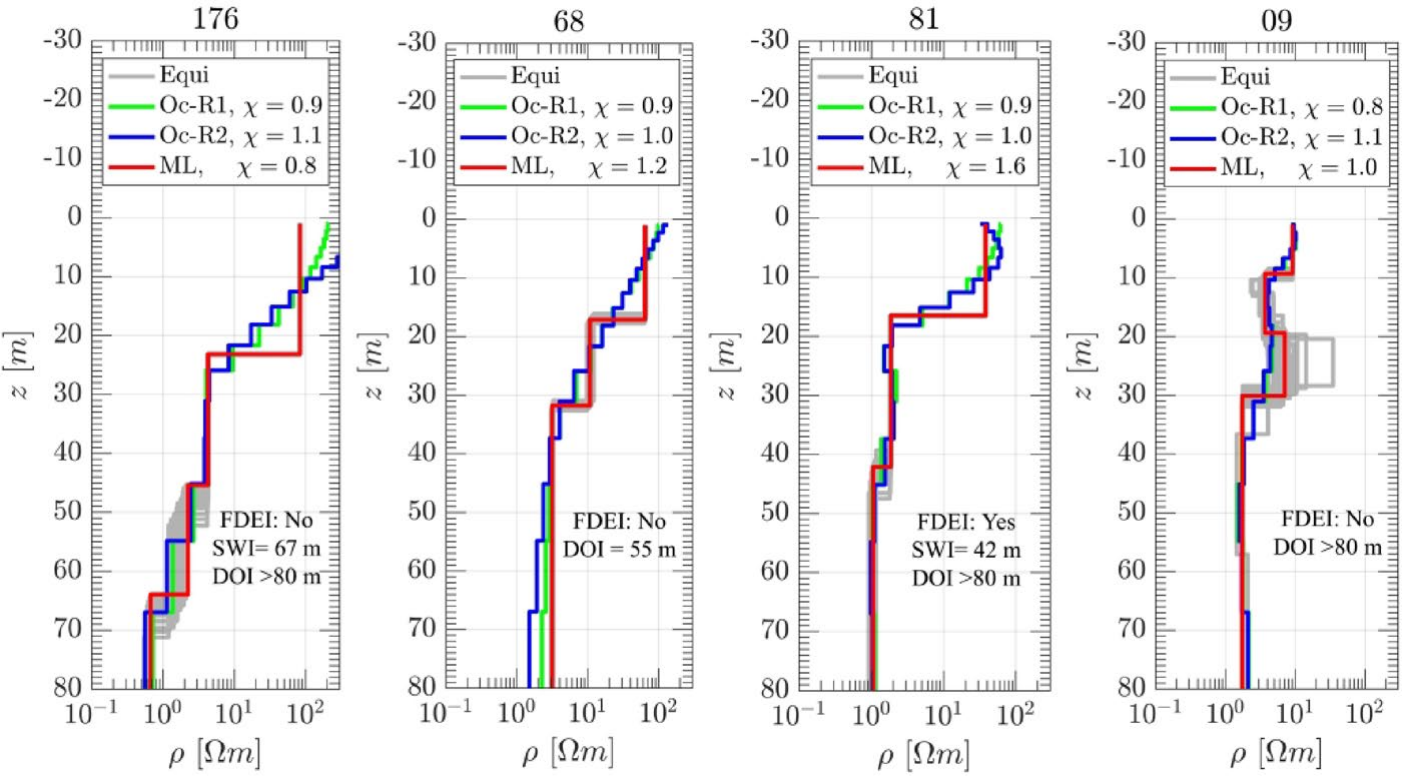
Inversion results of selected TEM sites (176, 68, 81, and 09) in a sparse data area (see locations in Fig. 7), used to evaluate the delineation of the FDEI boundary.

To address concerns regarding sparse data coverage in certain parts of the study area, Fig. 8 illustrates representative and high quality TEM soundings (sites 176, 68, 81, and 09; see their locations in Fig. 7) that fall within the zone where the FDEI was carefully delineated. The inversion results show contrast in DOI and saltwater interface detectability that directly affect the reliability of the FDEI interpretation. For example, site 176 clearly resolves the SWI at 67 m depth, with a DOI exceeding 80 m, yet it does not define the FDEI since it is not the farthest point along the BIN stream. In contrast, the adjacent site 68, despite its proximity, does not resolve the SWI due to limited depth coverage (DOI = 55 m), emphasizing a key uncertainty in extending the FDEI boundary. Similarly, site 81 detects the SWI at a depth of 42 m with sufficient DOI and considered as FDEI, while other locations to the east, such as site 09, lack clear interface identification despite having a DOI greater than 80 m.

The analysis of three identified patterns allowed us to explore and propose key characteristics that may explain the phenomena associated with the FDEI. For example, the concealed channel identified south of the BIN structural system appears to correlate with an eastern wadi on the terrain, marked by a yellow streamline (Fig. 7). This alignment suggests that the subsurface channel may serve as a conduit for brine migration, contributing to the observed eastward bulging trend of the saltwater interface by increasing soluble material and enhancing porosity. Furthermore, the BIN stream channel exhibits more pronounced landward saltwater intrusion compared to the MUT and MAZ channels. The simulation provided by Al-Halbouni et al.^8^ demonstrates that the hydrogeological response to the declining DS level is marked by a dynamic redistribution of groundwater flow and salinity. The hydrogeological simulation, conducted within a resistivity range of 0.1 to 100 Ωm (see Fig. 5 for resistivity scale comparison), illustrates how the falling DS level (40 m) leads to a steepening of the inland-directed hydraulic gradient, particularly near the retreating shoreline. This enhanced gradient facilitates the landward advance of SWI, which initially evolves slowly but becomes progressively more aggressive as subsurface conduits form, offering preferential flow paths. The BIN system appears to be more connected to such high-porosity pathways, allowing saltwater to penetrate farther inland than in the MUT and MAZ stream channels. Hence, these simulations offer a complementary perspective to the commonly accepted model of a retreating saltwater interface, by highlighting the role of density adjustment as a key mechanism controlling the SWI position in such stream channels.

Additionally, the spatial overlap between the FDEI and the sinkhole belt highlights a potential link between SWI and sinkhole distribution. The distribution of sinkholes across most surveyed sites suggests that the SWI is located within slightly saturated, brine-bearing sediments (0.3–1.0 Ωm) but not within fully saturated, brine-bearing sediments (< 0.3 Ωm), which are typically found closer to the lake^27^. However, subtle influences from fault or fracture systems cannot be entirely eliminated, as they may locally modulate these processes^9^.

### Saltwater intrusion interface: a stream-scale analysis in the region of Uvala (U1) and farms

**Fig. 9:**
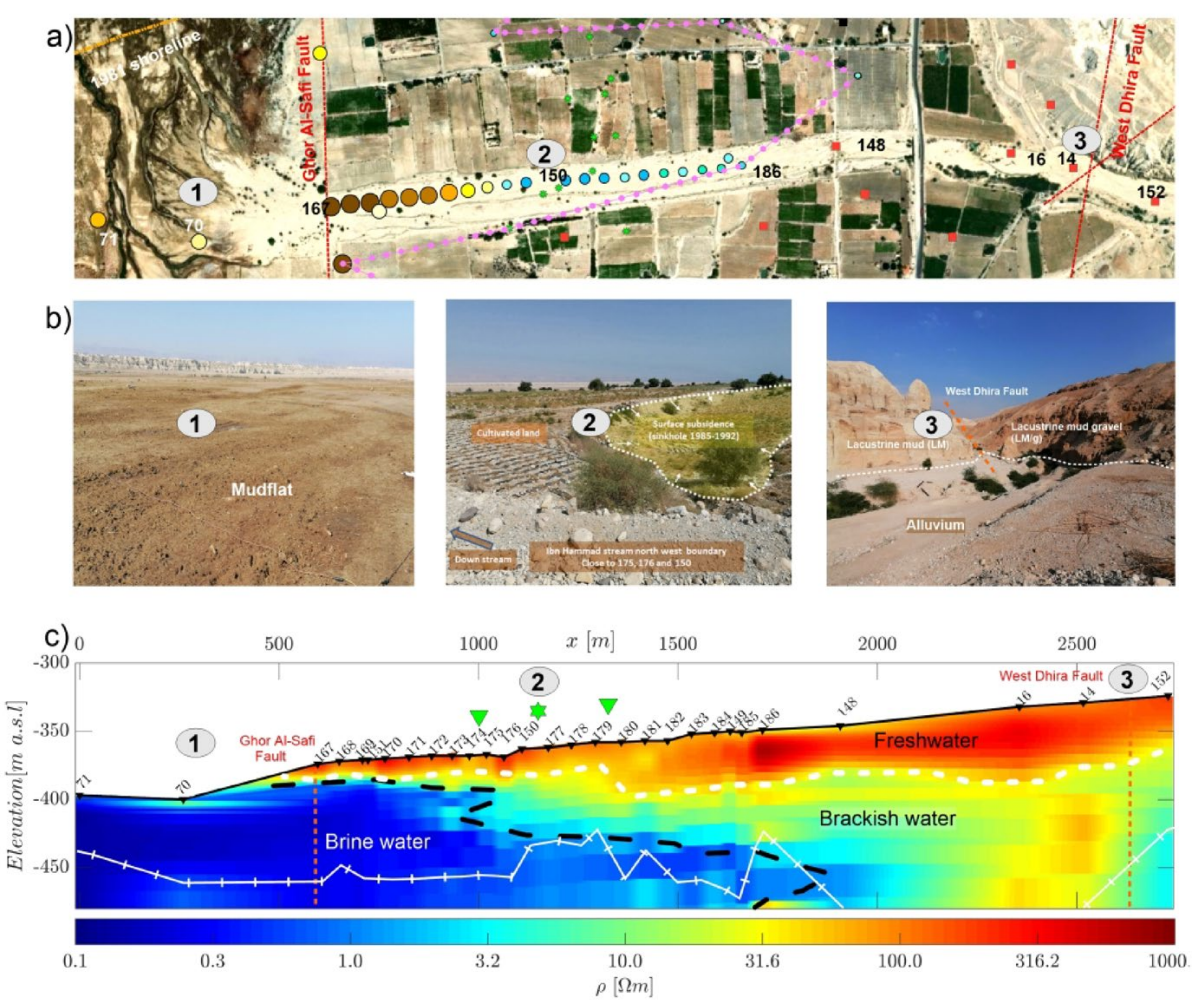
Structural interpretation of Wadi Ibn Hamad (BIN) resistivity model (**a**) Profile orientation and referenced TEM soundings. The pink line indicates the FDEI. See Fig. 7 for additional elements; (**b**) Field photo of three distinct topographic and structures features including upstream West Dhira Fault, surface depression near TEM soundings: 175, 176, and 150, the depression area is of 150 m^2^, downstream mudflat deposits; (**c**) Occam R1 resistivity model with interpreted resistivity and possible structural locations; the tick-dashed white line indicates the DOI.Green asterisk for sinkhole location and triangle for possible discontinuous/sedimentological structure.

The Wadi Ibn Hamad (BIN, Fig. 1c) serves as a major contributor to the area’s groundwater system, alongside water supplied from the Al-Karak Dam in the upstream area. Approximately 80% of the discharge at the nearby ‘Ain Maghara’ spring^8^ originates from base flow within the wadi, near the West Dhira Fault (Fig. 9a and b). The wadi supports a shallow clastic aquifer in a region characterized by intensive agricultural activity. At the downstream end, in the mudflat area, an alluvial fan has developed and has been significantly shaped by the base-level fall of the DS (Fig. 9c)^8^. Furthermore, sinkhole formation downstream, particularly in the region of U1 (cf. Fig. 1c), crosses the wadi and may influence groundwater flow within the system. Within the context of the available information, we analyze the geoelectrical characteristics of the BIN resistivity model as follows:

First, the saltwater interface, delineated as a dashed black line (Fig. 9c), demonstrates a smooth and gradual slope of approximately 4°, forming a wedge-like structure. Notably, many of the TEM sites have DOI that extends below this delineated interface, providing confidence in the resolution of deeper features. Second, the subsurface is stratified into three zones of potential clastic aquifers based on resistivity values (Table 1)^23^. The uppermost zone, comprising gravel and sand with resistivity values exceeding 80 Ωm, corresponds to a freshwater zone. The underlying sand-silt/clay zone, where resistivity progressively decreases from 30 Ωm to 2.0 Ωm, signifying brackish water or diluted freshwater. The lowest zone is characterized by saturated and unsaturated brines, consisting of clay, silt, and possible salt, with resistivity values < 1.0 Ωm. Remarkably, resistivity values < 0.3 Ωm near the mudflat area are indicative of saturated brine within sand or clay layers^27^.

Third, the area between soundings 171 and 181 exhibits unique sedimentological and structural features. Notably, (a) between 179 and 180 (x = 1300 m), the upper gravel layer undergoes an abrupt thinning towards sinkholes area, potentially indicating structural displacement. (b) another significant anomaly is observed at station 174 (x = 980 m), where the SWI assumes a complex “Z”-shaped pattern. This morphology is likely attributable to depositional and structural processes, such as the interfingering of alluvial deposits with an adjacent alluvial fan^13,29^. Furthermore, between (a) and (b) a refilled sinkhole (U1) is evident in the alluvial gravel area near stations 150, 175, and 176, intersecting the wadi system and depicted in Fig. 9a–c.

As a result, Fig. 9a and c emphasize the vulnerability of the Wadi Ibn Hamad and its surroundings to SWI, which exerts a profound impact on agricultural areas and water resources. TEM 148 marks the critical endpoint of the SWI along profile, with its inland extent reaching approximately 1.75 km and notably affecting northern adjacent agricultural lands (see pink line, Fig. 9a). Local observations suggest that the water table within the alluvial gravel-sand aquifer lies approximately 10 m below ground surface in the area of BIN downstream (communication with local farmers). Using the relationship Hs = 4.3×Hf^6^, where Hf represents the freshwater table elevation and Hs​ is the depth of the saltwater interface, Hs​ is estimated to be approximately 43 m. This estimate agrees well with the depth of the interface observed in the BIN model and aligns with the Ghyben-Herzberg approximation.

However, the classical Ghyben-Herzberg relationship assumes a seawater density of approximately 1.03 g/cm³, whereas DS water has a much higher density of about 1.23 g/cm³^3,6^. This larger density contrast reduces the depth of the saltwater interface for the same freshwater head, resulting in a much shallower and sharper transition zone. Moreover, due to the very low surface slope and hydraulic gradient in the study area, the interface tends to be more compressed and laterally extensive than in typical coastal aquifers. These combined effects are clearly reflected in our TEM results, where the transition is well-defined and located significantly farther inland. Furthermore, the BIN model shows that DS water intrudes farther inland than seawater under similar hydraulic conditions^6,8^, emphasizing the impact of both higher density and reduced hydraulic gradients. This finding has critical implications for groundwater resource management, especially in lowland areas adjacent to the DS.

Ultimately, for effective land management and SWI mitigation, new boreholes should align with the spatial distribution of the SWI map, optimally placed east of sounding 148 for monitoring or production while minimizing SWI risks.

## Conclusions

The transient electromagnetic (TEM) method was applied to map saltwater intrusion (SWI) from the Dead Sea (DS) in the Ghor Al-Hadith area (GAH). A total of 195 TEM soundings of multi-loop sizes were conducted, with higher density along stream channel systems, and spatially distributed for site-specific characteristics. TEM resistivity models provided insights into the subsurface geoelectrical structures, revealing a saltwater interface with resistivity values < 1.0 Ωm, detectable down to a depth of 100 m.

The potential effect of refilled sinkholes on TEM interpretation was evaluated in the region of borehole BH2. In areas such as uvala 1 (U1), where several refilled sinkholes are present, no significant distortions were observed in the resistivity models. These features, along with the active hollow sinkholes in uvala 2 (U2), are considered shallow topographic variations rather than true three-dimensional resistive bodies. U2 lies beyond the inland extent of the current SWI and was sufficiently interpreted using a one-dimensional (1D) approach and remains outside the scope of the present study.

Mapping the saltwater interface consistently across all sites is challenging due to local variations in depth of investigation (DOI) and geological conditions. However, this study shows good agreement with previous simulation results based on a 40 m decline in the DS water level. The observed inland extent of the SWI matches the simulated pattern, supporting the idea that density-driven processes influence salinity distribution.

It is important to note that some subareas of GAH may deviate from the classical Ghyben-Herzberg model, which assumes hydrostatic conditions and a sharp interface, due to geological complexity, including conduit networks and possible upwelling of saline water from deeper layers through faults, resulting in a more dynamic and variable SWI behavior. The comparison findings also demonstrate that SWI mapping depends on the scale of the survey. While the TEM data reveal the broader structure, smaller features such as narrow stream channels and karstic voids may remain undetected, adding uncertainty to the interpretation of local SWI at various depths. Notably, the depth to the saltwater interface appears to follow the Ibn Hamad stream channel (BIN) and a possible concealed stream channel to its south in the central area. In contrast, at other sites, the interface depth seems constrained by the sinkhole belt, with no clear correlation to the NNW–SSE strike-slip faults. The intrusion is bounded to the north and south by the Mutayl (MUT) and Al-Mazra’a (MAZ) stream channels, respectively, while the central area shows a more pronounced inland advance, with the intrusion extending 1.75 km inland relative to the 1981 shoreline.

The resistivity models, particularly from the BIN stream channel, allowed for detailed differentiation of subsurface clastic aquifers, which consisting of three distinct zones. These zones become clear in land, reflecting varying salinity levels, with the uppermost zone corresponding to a freshwater aquifer (> 80 Ωm), an intermediate zone indicating a brackish aquifer (2.0–30 Ωm), and the deepest zone associated with DS brine of varying saturation (< 1.0 Ωm). The models further demonstrate their impact on agricultural areas and water resources, aiding in the understanding of aquifer vulnerability to SWI and informing water management strategies in the GAH area. These integrated observations provide the first spatially comprehensive characterization of the contemporary configuration of the SWI, establishing a critical baseline for evaluating its temporal dynamics and future evolution.

## Data Availability

The datasets used and/or analysed during the current study available from the corresponding author on reasonable request.
